# Maize hydroxycinnamic acids: unveiling their role in stress resilience and human health

**DOI:** 10.3389/fnut.2024.1322904

**Published:** 2024-02-02

**Authors:** Tzitziki González-Rodríguez, Silverio García-Lara

**Affiliations:** Tecnologico de Monterrey, School of Engineering and Science, Monterrey, Nuevo León, Mexico

**Keywords:** maize, bioactive compounds, breeding, hydroxycinnamic acid amides, plant defense, phenolics

## Abstract

Maize production is pivotal in ensuring food security, particularly in developing countries. However, the crop encounters multiple challenges stemming from climatic changes that adversely affect its yield, including biotic and abiotic stresses during production and storage. A promising strategy for enhancing maize resilience to these challenges involves modulating its hydroxycinnamic acid amides (HCAAs) content. HCAAs are secondary metabolites present in plants that are essential in developmental processes, substantially contributing to defense mechanisms against environmental stressors, pests, and pathogens, and exhibiting beneficial effects on human health. This mini-review aims to provide a comprehensive overview of HCAAs in maize, including their biosynthesis, functions, distribution, and health potential applications.

## 1 Introduction

Maize (*Zea mays L.*) stands as one of the most critical staple crops worldwide, providing sustenance for billions of people. However, maize production encounters several challenges, including biotic and abiotic stresses that can substantially impact yield and global food security (1). One promising avenue to address these challenges is the modulation of bioactive compounds.

**Figure 1:**
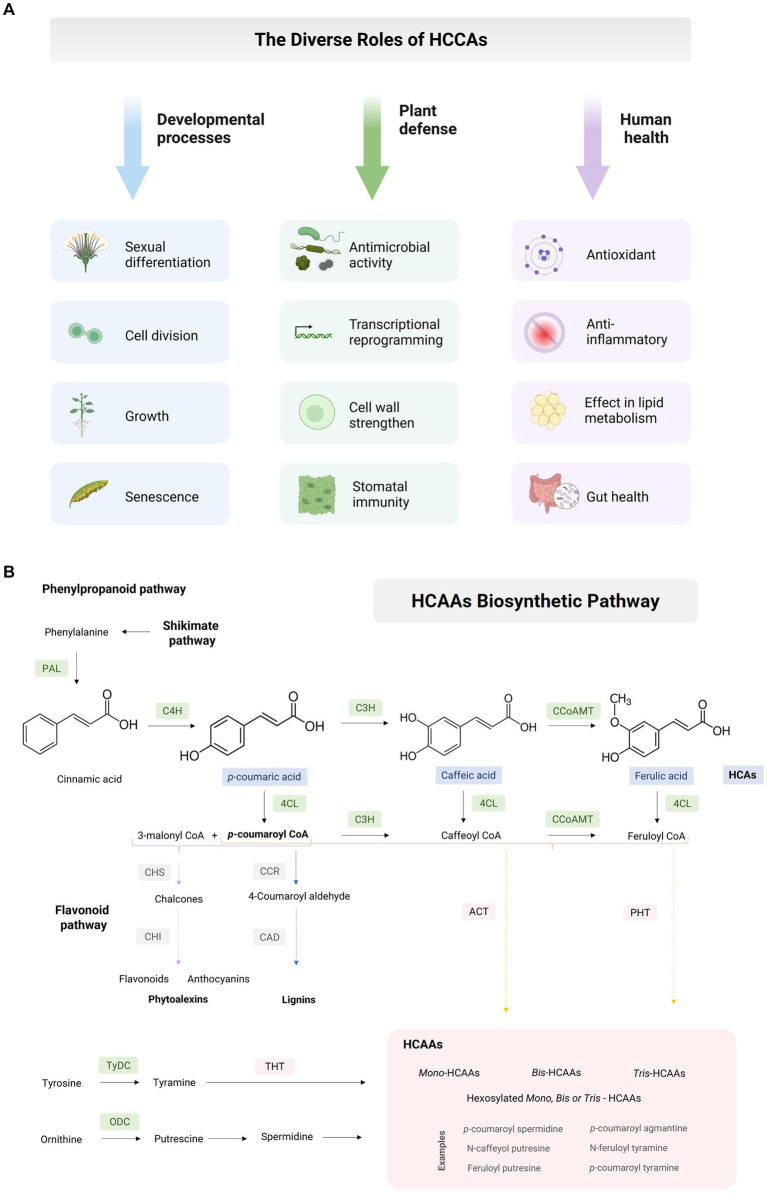
Maize hydroxycinnamic acid amides. **(A)** The diverse roles of plant hydroxycinnamic acid amides in the developmental processes, defense responses, and impact on human health. **(B)** Hydroxycinnamic acid amides (HCAAs) biosynthetic pathway. Phenylpropanoid pathway (PPP): Phenylalanine ammonia-lyase (PAL), cinnamate 4-hydroxylase (C4H), cinnamate 3-hydrolase (C3H), 4-coumaroyl-coenzyme A ligase (4CL), caffeoyl-CoA o-methyltransferase (CCoAOMT). Flavonoid pathway: Chalcone synthase (CHS), chalcone isomerase (CHI). Lignins: Cinnamoyl CoA reductase (CCR), cinnamyl alcohol dehydrogenase (CAD). HCAAs: Agmatine *N-*coumaryl transferase (ACT), putrescine hydroxycinnamoyl transferase (PHT), tyramine *N*-hydroxycinnamoyl transferase (THT). Amino acids: tyrosine decarboxylase (TyDC), ornithine decarboxylase (ODC).

Hydroxycinnamic acid amides (HCAAs) constitute a group of secondary metabolites widely distributed across the plant kingdom, with roles extending to developmental processes including sexual differentiation, cell division, growth, and senescence (2). HCAAs also assume significance in plant stress responses, including defense against insect herbivory, resistance to multiple pathogen infections (3), and resilience to abiotic stresses such as drought, salinity, and mechanical injury (4–7). As valuable antioxidants, HCAAs also hold the potential to mitigate various chronic human diseases (8–13) (Figure 1A). Nevertheless, the specific roles of HCAAs in maize are still not completely understood.

HCAAs represent polymers synthesized through the condensation of hydroxycinnamic acid (HCA) and either mono- or polyamines (PAs) via the phenylpropanoid pathway (PPP). They derive from phenylalanine and tyrosine and possess a C3–C6 carbon skeleton with a series of hydroxylations and methylations on the aromatic ring, resulting in diverse structural patterns (14). The process initiates with the enzyme phenylalanine ammonia-lyase (PAL), which catalyzes the conversion of phenylalanine into trans-cinnamic acid. Subsequently, cinnamate 4-hydroxylase (C4H) transforms cinnamate into *p-*coumaric acid, and 4-coumarate CoA ligase (4CL) converts it into *p-*coumaroyl CoA. These reactions constitute a critical step in the PPP, responsible for the biosynthesis of various secondary metabolites, including flavonoids, anthocyanins, lignin, and other compounds (Figure 1B).

Understanding the genetic mechanisms controlling HCAA production in maize becomes vital. Genome-wide association studies (GWAS) and quantitative trait loci analyses (QTL), coupled with untargeted metabolomics have revealed genes regulating HCAA biosynthesis. Studies have shown disparities in HCAA abundances among cultivars, which are further affected by environmental factors such as growing locations and seasons, serving as dominant parameters driving metabolite profile variability in corn (15, 16). GWAS and QTL analysis in kernels and leaves have elucidated the genetic architecture of these traits in various maize inbred lines. Metabolomics analyses have unveiled a wide range of metabolic variations in analyzed populations, identifying key candidate genes responsible for the novel metabolites involved in phenolic and HCAA formation (17–19). However, limited efforts have been directed toward increasing their concentration in maize (20).

The most prevalent HCA derivatives in maize include *p*-coumaric, ferulic, caffeic, and sinapic acids. Research suggests that HCAs offer several agronomic advantages, and their content can potentially be enhanced via conventional or biotechnological breeding. Such enhancement could improve the resistance of the crop to environmental and storage-related stresses, and find applications in natural dietary supplements, functional ingredients, and active additives for cosmetics (15, 21).

Despite its paramount significance as a crop, further research is warranted to comprehend metabolic disparities among maize varieties and to explore the chemical diversity, abundance, and biological properties of maize HCAAs. This review summarizes current knowledge regarding this compound family in maize, including its role in stress responses and its potential for conferring health benefits to humans.

## 2 Diversity of HCAAs in maize tissues and genotypes

Maize, extensively studied for its nutritional benefits, contains bioactive compounds, dietary fiber, vitamin A, and lysine. In comparison to other cereals, exhibits the highest levels of bound ferulic acid and total phenolics (22). Sweet maize is distinguished by its higher free phenolic and flavonoid content than regular corn. Various corn varieties exhibit ferulic and *p*-coumaric acids as the predominant phenolic compounds. Pigmented maize varieties, such as blue and purple, showcase a diverse variety of phenolic acids and anthocyanins not commonly found in their yellow counterparts (23). Some flavonoids, including eriodictyol, luteolin, isoorientin, and maysin, are prevalent in pollen, silk, and tassel of maize but are less frequently detected in the seeds. Although certain flavanol-anthocyanins have been identified in maize, their quantification remains to be studied. The accumulation of phenolic acids, including gallic acid, chlorogenic acid, syringic acid, and HCA, can be attributed to the expression of PAL during cultivation (16). Additionally, cell wall-bound phenolics, such as ferulic acid, may be associated with specific tissue morphological structures (24).

Notably, nearly four decades ago, an abundance of peculiar conjugates was discovered in the male reproductive organs of maize, as reported by Martin-Tanguy et al. in 1978. These conjugates were identified as hydroxycinnamic acid amides, with ferulic acid specifically linked to the individual amino groups of putrescine and spermidine (25). Despite their discovery long ago, little research has reported their role in maize.

HCAAs represent a group of conjugated PAs, encompassing cinnamic acid, coumaric acid, caffeic acid, ferulic acid, and sinapic acid, which combine to form acylated PAs (10, 26). HCAAs predominantly exist as esters located within subcellular compartments such as vacuoles, the cell wall, or the cytosol (3). Numerous HCAs and HCAAs have been previously identified in maize genotypes of diverse origins.

The highest concentrations of HCAAs were detected in the outer tissues of the maize kernel, specifically in the pericarp and aleurone fraction, constituting up to 75% of the total HCAA content in the grain. The germ contained the second-highest concentration of HCAAs, whereas nearly none were detected in the endosperm (15). Alternatively, these phenolic amides are found as co-pigments in colored maize and contribute significantly to anthocyanin coloration, particularly putrescines for red and spermidine for blue-colored varieties (27). HCAAs not only exhibit variations between genotypes, kernel colors, and ecological factors but also vary across different stages of plant development (11).

Diferuloylputrescine and *p*-coumaroyl-feruloylputrescine were identified as abundant PA conjugates in lipid extracts of maize kernels (28). Subsequent variations of these compounds have been associated with genetic sources, varieties, and altitude of origin. Maize varieties with elevated HCAA concentrations were preferentially selected at lower altitudes (15). In such cases, the pericarp and aleurone layers exhibited a higher HCAA concentration. This highlights the significant potential of key genes in the HCAA pathway for application in stress resistance breeding strategies.

A recent study evaluated 66 hybrids for hydroxycinnamic acid concentration in the grain, along with field yield and test weight. The findings suggest that breeding maize for improved HCA concentration is not only feasible but could also facilitate the production of dietary supplements or natural food additives while enhancing resistance to biotic and abiotic stresses during both the growing season and grain storage (20).

## 3 Role of HCAAs in maize response to stress

Plants have harnessed the utility of hydroxycinnamic acid amides in combating biotic stress and adapting to environmental changes. These compounds can activate defense genes, strengthen cell walls, regulate oxidative stress, and promote lignin accumulation (Figure 1A). Consequently, HCAAs play a crucial role in the survival and growth of plants under challenging conditions. While a comprehensive understanding of the general function of HCAAs in plant immunity is available elsewhere (3), this discussion will focus on their role in maize responses to stress.

### 3.1 Transcriptional regulation of defense genes

One of the mechanisms plants employ in response to stress signals is the transcriptional regulation of genes involved in secondary metabolic synthesis. In maize, there is strong evidence of the upregulation of the HCAA pathway following *Puccinia sorghi* infection. Genes associated with defense responses and secondary metabolism, such as those related to the phenylpropanoid, flavonoid, and terpenoid pathways, are induced in response to this stress. Metabolome analyses have confirmed the accumulation of chlorogenic acid, caffeic acid, and ferulic acid in all lines analyzed, along with an increase in coumaric acid and its flavonoid derivatives in resistant lines (21). Families of transcription factors, including WRKY and MYB, have been reported to regulate the biosynthesis of secondary metabolites, including HCAAs (29). For instance, the MYB transcription factor *yellow seed 1* is associated with maize protection against *Colletotrichum sublineolum* infection (30).

### 3.2 Cell wall properties

Recent studies suggest that HCAAs are bound to the arabinoxylans of the cell wall, leading to increased cell wall thickness and enhanced strength. This effect serves to restrict the penetration and infection of pathogens (3). Several reports also have revealed the importance of these compounds in postharvest insect resistance. Phenolic compounds bound to the cell wall are involved in the resistance of maize against the Mediterranean corn borer *Sesamia nonagrioides*. Researchers evaluated various maize genotypes with differing HCA contents and borer resistance levels. They also investigated the relationships between several cell wall-bound phenolic compounds, including ferulic acid and its dimers, *p*-coumaric acid, and syringyl lignin subunits. The results showed significant correlations between both ferulic acid and its dimers and *p*-coumaric acid with the damage inflicted *by S. nonagrioides* larvae, as measured by tunnel lengths (31).

Another study explored the contribution of cell wall components in the pericarp to resistance against the maize weevil (*Sitophilus zeamais*) across nine different genotypes of tropical maize. The study examined six parameters related to susceptibility to the weevil, including measurements of certain HCAs. The results indicated that cell wall cross-linking components play a role in enhancing kernel resistance against *S. zeamais* (32). Substantial progress has been made through recurrent selection and the improvement of a maize population against the maize weevil and the larger grain borer (*Prostephanus truncatus*). Comparison of the phytochemical composition of the pericarp cell wall before and after selection revealed a 42% increase in cell wall-bound components, including ferulic and diferulic acids. Moreover, the endosperm exhibited an 18% increase in free phenolic acid (33).

Maize cell wall secondary metabolites also play a role in abiotic stress responses. Recent findings have revealed how cell walls undergo remodeling in response to salinity stress. Cell wall secondary metabolites, particularly cellulose, matrix polysaccharides, and lignin, are affected by salt stress in both the roots and stems of seedlings and mature plants. Furthermore, the expression of genes and the activity of enzymes involved in PPP biosynthesis increase under salt stress conditions. Also, metabolite profiling has confirmed the accumulation of secondary metabolites in response to salinity stress (6). Similarly, plant cell walls can be strengthened by lignin and callose deposition. Lignin, predominantly deposited in secondary cell walls, acts as a physical barrier to limit the spread of pathogens (34). On the other hand, callose deposition can prevent pathogens from entering epidermal cells. HCAAs can enhance the synthesis of lignin and the deposition of callose (35).

### 3.3 Emerging functions and roles

Plants produce hormones that aid in their defense against pathogens and induce disease resistance. The hormone jasmonic acid (JA) regulates the biosynthesis of secondary compounds, including HCAAs (21). The accumulation of HCAAs is induced by JA and ethylene signals, whereas salicylic acid (SA) plays a crucial role in initiating plant defense. The introduction of SA stimulates the production of ferulic acid, *p*-coumaric acid, and sinapic acid, which enhances plant resistance. Then, *p*-coumaric acid can trigger JA signaling-mediated induction of phenylpropanoid biosynthesis, contributing to disease resistance (3).

The accumulation of HCAAs also improves the resistance of maize to multiple pathogens, including fungi such as *Aspergillus flavus* (36, 37) and *Fusarium* (38), as well as herbivores including *Spodoptera littoralis* (39), and the aforementioned insects.

Maize genotypes exhibit varying levels of HCAAs and present a valuable resource for targeted breeding programs aimed at developing resilient cultivars (15). Moreover, the underexplored genetic diversity within maize, notably in local landraces, offers an avenue for discovering novel traits that can be incorporated into modern varieties. Furthermore, the presence of various compounds in pigmented maize varieties adds another layer of value, with potential applications for human health (23, 40, 41).

## 4 Maize-derived HCAAs: implications for health

The HCAA family exhibits a wide array of biological activities, ranging from antifungal and antimicrobial properties to anti-inflammatory and anticancer properties (Figure 1A). Given the abundance of these compounds in various food sources, there exists an opportunity to explore their chemical diversity and develop analogs with enhanced potency. Notably, HCAAs may also be present in maize-derived products, such as cornbread, commonly known as *broa* (42, 43). Evidence suggests that diferuloylputrescine, isolated from corn bran, has demonstrated efficacy in inducing apoptosis in human leukemia U937 cells (44). While HCAA quantification demands the utilization of multiple analytical techniques, recent progress in this field has rendered the discovery of new conjugates with potential health benefits more attainable (45).

### 4.1 Antioxidant and anti-inflammatory properties

HCAA molecules are distinguished by their heightened antioxidant activity, attributed to the presence of amide species, which also enhances their stability under physiological conditions and during delivery methods. Unlike esters, which can be readily broken down by the hydrolase enzymes in the human body, amides are better suited for oral administration, offering potential anti-inflammatory (46), antioxidant (16), and antimelanogenic benefits (9, 47).

The antioxidant capabilities of corn bran have been demonstrated via three distinct *in vitro* assays, involving mushroom tyrosinase and B16 melanoma cells. *N,N*‘-dicoumaroyl-putrescine, *N*-*p*-coumaroyl-*N*‘-feruloylputrescine, *N*,*N*‘-diferuloyl-putrescine, and their related HCAs, *p*-coumaric acid and ferulic acid, have demonstrated melanogenesis inhibitory activity. These compounds exhibit the potential to serve as natural antioxidants and skin-whitening agents (9).

### 4.2 Implications in gut health

Diverse studies have revealed the positive effects of HCAs on gut health (48). For instance, the Peruvian purple maize variety AREQ-084, distinguished by its high hydroxycinnamic acids and dietary fiber content, also contains phenolic components such as anthocyanins, which exhibit potential health benefits. These bioactive compounds promote gut health by influencing the activity of beneficial probiotic bacteria like *Lactobacillus helveticus* and *Bifidobacterium longum,* but without adversely affecting the pathogenic *Helicobacter pylori*. The anthocyanin-linked coloring properties of purple maize can be harnessed in the development of probiotic-functional foods, leading to new avenues for improving gut health (49, 50).

### 4.3 Effect on lipid metabolism

Research indicates that HCAA molecules may play a pivotal role in regulating diseases associated with metabolic syndrome, including obesity, diabetes, insulin resistance, and hypertension. HCAAs derived from corn, such as *N*-*p*-coumaroyl-*N*‘-feruloylputrescine and *N*,*N*‘-diferuloylputrescine, exhibit inhibitory effects on α-glucosidase, an enzyme responsible for catalyzing the final step in dietary carbohydrate digestion, leading to the suppression of post-meal glucose levels (49, 51) also demonstrated the potential health benefits of HCAAs via *in vitro* assay models targeting hyperglycemia (α-glucosidase and α-amylase inhibition) and obesity (lipase inhibition). These findings suggest that HCAAs may serve as promising candidates for the regulation of lipid metabolism and related disorders, potentially mitigating the adverse effects associated with obesity (8).

## 5 Current analytical challenges

Metabolomics in maize has significantly improved in recent years, enabling the quantification and profiling of a wide range of compounds (18). However, due to the extensive diversity in plant metabolism, it is nearly impossible to comprehensively determine the metabolome using a single protocol. In recent years, research has been conducted to identify and quantify HCAAs in various tissues of maize genotypes, utilizing diverse analytical techniques (Table 1).

**Table 1:** Research conducted in the past 5 years whereby HCAAs were identified and/or quantified in different tissues of maize genotypes by using different analytical techniques.

Genotypes	Tissue	Purpose	Analytical technique	Compounds	Reference
White, red, and orange maize from race *Cabanita*	Kernels	Characterization of primary and secondary metabolites at different maturity stages and *in vitro* analysis for health-related properties	UHPLC	*p*-coumaric acid derivatives, ferulic acid derivatives, total HCA	(11)
IBM RIL population and parental strains	Leaves and whole seed	QTL mapping of metabolites through an untargeted approach	DLI-ESI MS	Feruloyl putrescine, coumaroyl putrescine, *N*(1), *N*(8)-bis(coumaroyl)spermidine, *N*,*N*′-bis-(*p*-coumaroyl)-*N*″-feruloyl spermidine	(17)
Near-isogenic lines H95 and H95:Rp1-D	Seedlings	To understand the resistance mechanisms against *Puccinia sorghi*	UHPLC-HRMS	4-coumaric acid, caffeic acid, ferulic acid, sinapic acid, chlorogenic acid	(21)
32 Mexican landraces	Pericarp, endosperm, and germ	To determine the content and localization of HCAA in maize kernels	HPLC-PDA	Diferuloyl putrescine, feruloyl putrescine, cinnamoyl putrescine, caffeoyl putrescine and coumaroyl putrescine	(15)
Maize seeds (Unspecified genotype)	Whole seed	Creation of a novel and complete database of HCAAs	UHPLC-HRMS	*N*,*N*′-bis-feruloyl-putrescine, *N*,*N*′-(*p*-coumaroyl)-feruloyl-putrescine, *N*,*N*′-bis-(*p*-coumaroyl)-spermidine *N*,*N*′-(*p*-coumaroyl)-feruloyl-spermidine and *N*-trans-feruloyl-putrescine	(14)

UHPLC, Ultra high-performance liquid chromatography; DLI, Direct liquid introduction; ESI, Electrospray ionization; MS, Mass spectrometry; HRMS: High-resolution MS; HPLC, High-performance liquid chromatography; PDA, Photodiode array.

The analysis of HCAAs is conducted through various methods, including liquid chromatography (LC) and LC coupled with mass spectrometry (LC–MS). Nevertheless, despite numerous attempts, the distribution of HCAAs has only been identified within specific tissues and maize genotypes. Moreover, annotating HCAAs from untargeted metabolomics data poses challenges due to the limited availability of authentic commercial references and the absence of HCAA entries in existing MS databases (14). A novel method for detecting plant HCAAs is needed to comprehensively analyze all types of HCAAs.

Due to the absence of accurate methodologies, Li et al. (14) devised a comprehensive workflow for the deep annotation of HCAAs using UHPLC-high resolution MS (UHPLC-HRMS) in conjunction with an *in-silico* database of HCAAs. This *in-silico* database was constructed including 846 HCAAs derived from common phenolic acids and PA/aromatic monoamine substrates, representing potential biosynthetic pathways for plant-specialized HCAA structures. To establish characteristic MS/MS fragmentation patterns of HCAAs, reference mixtures were used. This comprehensive study successfully identified a total of 79 HCAAs, including 42 compounds newly identified in maize, wheat, and rice, and 20 that have never been reported to exist in plants. These results highlight the potential of the developed method to identify HCAAs in plants even in the absence of prior knowledge regarding HCAA distributions (14).

Furthermore, recent advances in analytical protocols have facilitated the rapid detection of HCAA compounds through direct injection into a mass spectrometer. These compounds exhibit distinctive fragmentation patterns during MS analysis, significantly simplifying the annotation of metabolites (52, 53). However, modern techniques need to be explored for the precise quantification of molecules in maize tissues. Recent strides in analytical instrumentation, including infrared spectroscopy (54) and ambient mass spectrometry (55), have the potential to revolutionize our capacity to analyze the molecular composition of maize plants, offering exciting prospects for agricultural research and crop improvement.

## 6 Discussion and conclusion

The current knowledge about HCAAs in maize is limited, particularly regarding their distribution, concentrations, and diversity across maize germplasm. Despite its abundance, maize diversity remains understudied. The greatest diversity of maize germplasm resides in its center of origin, Mexico, where over 60 maize landraces have been documented (56). These genetic resources hold immense potential for identifying novel, advantageous traits that can be introgressed into modern maize varieties, enhancing their adaptation to climatic change. Although the biosynthetic pathway of HCAAs is known, a comprehensive study of novel HCAAs is still lacking. It is also unclear whether HCAAs are solely storage compounds or actively functional.

Compared to other plants (3, 35) the lack of knowledge about HCAAs in maize emphasizes the need for dedicated efforts to expand databases and conduct integrative multi-omic studies. These studies could help uncover the diversity of HCAAs and identify their production mechanism and genotypes with higher concentrations. Addressing these gaps is crucial for formulating targeted breeding strategies to increase HCAA levels due to the pivotal roles these compounds play in plant defense responses. This can involve harnessing the natural genetic diversity of maize to identify and develop elite cultivars enriched in compounds. Moreover, genetic engineering techniques such as CRISPR/Cas could offer opportunities to manipulate and increase HCAA content (57).

Current advancements in analytical methods have resulted in the identification and synthesis of a wide range of HCAA standards (14). However, studies focused on identifying potential or novel HCAAs in maize have been scarce (18). To address these limitations, future research should prioritize the elucidation of HCAA structures, interactions, and synergistic effects with other bioactive compounds. The combined application of analytical methods and technologies, including high-throughput metabolomics coupled with advancements in computer science fields (data mining, machine learning, etc.) can contribute to more precise, cost-effective, and efficient studies (58).

HCAAs, known for their antioxidant and anti-inflammatory properties, also emerge as a promising resource for various therapeutic human applications. Their potential to positively influence gut health and lipid metabolism offers significant prospects for enhancing human well-being. However, their potential requires further animal studies and clinical trials to understand their safety and efficacy.

Exploring the natural genetic diversity of maize and harnessing elite cultivars enriched in beneficial compounds stands as a promising approach to elevating HCAA content. This not only contributes to agricultural advancements but also opens doors to understanding the functional roles of these compounds within maize, impacting both crop production and potential human health benefits.

## Author contributions

SG-L: Investigation, Methodology, Resources, Supervision, Validation, Writing – review & editing. TG-R: Conceptualization, Formal analysis, Methodology, Writing – original draft.
